# Phylogeography of a Habitat Specialist with High Dispersal Capability: The Savi’s Warbler *Locustella luscinioides*

**DOI:** 10.1371/journal.pone.0038497

**Published:** 2012-06-11

**Authors:** Júlio M. Neto, José L. Arroyo, Bruno Bargain, Juan S. Monrós, Norbert Mátrai, Petr Procházka, Pavel Zehtindjiev

**Affiliations:** 1 Department of Biology, University of Lund, Ecology Building, Lund, Sweden; 2 CIBIO/UP-Centro de Investigação em Biodiversidade e Recursos Genéticos, Universidade do Porto, Campus Agrário de Vairão, Vairão, Portugal; 3 Grupo Ibérico de Anillamiento, Fuentes de Nava, Palencia, Spain; 4 Bretagne-Vivante, Village de Trunvel, Tréogat, France; 5 Institute ‘‘Cavanilles’’ of Biodiversity and Evolutionary Biology, University of Valencia, Valencia, Spain; 6 Department of Zoology and Animal Ecology, Szent István University, Gödöllő, Hungary; 7 Institute of Vertebrate Biology, Academy of Sciences of the Czech Republic, Brno, Czech Republic; 8 Institute of Biodiversity and Ecosystem Research, Bulgarian Academy of Sciences, Sofia, Bulgaria; Biodiversity Insitute of Ontario - University of Guelph, Canada

## Abstract

In order to describe the influence of Pleistocene glaciations on the genetic structure and demography of a highly mobile, but specialized, passerine, the Savi’s Warbler (*Locustella luscinioides*), mitochondrial DNA sequences (ND2) and microsatellites were analysed in c.330 individuals of 17 breeding and two wintering populations. Phylogenetic, population genetics and coalescent methods were used to describe the genetic structure, determine the timing of the major splits and model the demography of populations. Savi’s Warblers split from its sister species c.8 million years ago and have two major haplotype groups that diverged in the early/middle Pleistocene. One of these clades originated in the Balkans and is currently widespread, showing strong evidence for population expansion; whereas the other is restricted to Iberia and remained stable. Microsatellites agreed with a genetic break around the Pyrenees, but showed considerable introgression and a weaker genetic structure. Both genetic markers showed an isolation-by-distance pattern associated with the population expansion of the eastern clade. Breeding populations seem to be segregated at the wintering sites, but results on migratory connectivity are preliminary. Savi’s Warbler is the only known migratory bird species in which Iberian birds did not expand beyond the Pyrenees after the last glaciation. Despite the long period of independent evolution of western and eastern populations, complete introgression occurred when these groups met in Iberia. Mitochondrial sequences indicated the existence of refugia-within-refugia in the Iberian Peninsula during the last glacial period, which is surprising given the high dispersal capacity of this species. Plumage differences of eastern subspecies seemed to have evolved recently through natural selection, in agreement with the glacial expansion hypothesis. This study supports the great importance of the Iberian Peninsula and its role for the conservation of genetic variation.

## Introduction

Historic events, such as changes in climate and topography, have been shown to leave genetic signatures that subsist to the present time [1], [2]. The quaternary glaciations, in particular, are thought to have been a major driving force of diversification and speciation, being the subject of multiple studies [3], [4]. During this period, most species have shown evidence for demographic and distributional changes (expansions and contractions) that seemed to occur in syntony with the advance and retreat of the ice sheets during the glacial cycles [5]. Temperate species reached their minimum areas of distribution (i.e. refugia as defined by Stewart et al [6]) during the glacial maxima, and the location of these refugia is generally consistent across species [1], [2]. In Europe, three discrete major refugia were identified: the Iberian Peninsula, the Italian Peninsula and the Balkan region, which remained ice free and led to independent, allopatric evolution for considerable periods of time [1], [2], [6]. Populations expanding from these refugia often met at suture (or hybrid) zones that are also consistent across species, for instance at the Pyrenees and the Alps, across central Europe and across central Scandinavia [2].

However, species-specific patterns are found in the timing of divergence between major genetic lineages, in the number and location of refugia as well as on post-glacial colonization routes, even if only temperate species are considered [2], [7]. In addition, recent detailed analysis of the geographic distribution of genetic variation revealed the existence of refugia within refugia [8], [9], especially in species with low-dispersal capabilities; and also of micro-refugia (which are located further north in temperate species) in areas where the climate during the last glaciations was mild enough to permit the survival of small populations [6], . In order to determine the factors associated with the variation in the phylogeographic patterns, a large number of species with a diversity of ecologies should be studied using multiple genetic markers and appropriate statistical approaches [4].

In this study, we describe for the first time the phylogeography of the Savi’s Warbler (*Locustella luscinioides*), a small, insectivorous passerine that breeds exclusively in medium to large reed beds across the Palaearctic, and winters along a narrow belt in sub-Saharan Africa [11]. Its breeding distribution is split into two areas: (1) from Western Europe to the west coast of the Black Sea, where the nominate subspecies occurs, and (2) from Kazakhstan to central China, were the *L. l. fusca* occurs. A third subspecies (*L. l. sarmatica*) is sometimes recognized, occurring from the Sea of Azov to the Black Sea (i.e. on the southeast part of the western range). These subspecies were described on the basis of subtle plumage colour differences, with eastern birds having paler underparts, olive rather than brown upperparts, and more obvious white tips to under-tail coverts [11].

Savi’s Warblers favour mosaics of marsh habitats with plenty of reeds, which provide both food and nest sites [12]. Its distribution in south and west Europe is scattered, being absent from apparently suitable habitats, and due to its decline and/or very localized distribution, this species is considered vulnerable or even threatened in some countries (e.g. Portugal, Spain [13], [14]). On the other hand, the distribution in central Europe and eastern countries seems to be more continuous (although this could result from lack of detailed information), having very large populations especially in Romania, but also further east. Some of the Baltic countries have been recently colonized, where the populations are currently increasing [11].

We analysed the phylogeography and population genetics of birds belonging to 17 breeding populations, plus a few birds from two wintering sites. Our purpose was to: (1) evaluate the mitochondrial and nuclear genetic structure, identifying evolutionary units that could potentially be of conservation value; (2) determine the timing of divergence of the Savi’s Warbler from its sister species (the River Warbler, *L. fluviatilis*) and between its major genetic lineages, thereby assessing the importance of the Pleistocene glaciations to a specialized, naturally-fragmented species; and (3) identify the number and location of refugia within Europe, as well as the major post-glacial expansion routes, comparing with other species.

## Methods

### Sampling and Laboratory Analysis

Blood samples of Savi’s Warblers were obtained from individuals belonging to 17 breeding populations across Europe and two wintering populations in sub-Saharan Africa (see Table 1). The samples were obtained during normal ringing procedures, by puncturing the wing vein and storing the blood in 95% ethanol and the birds were subsequently released, except for the two birds from Tuva, Russia, which were collected. In order to avoid sampling transient migrants, blood was only collected during the breeding season or, in case of adults and for some sites, during the post-breeding moult, which usually occurs close to the breeding territories [15].

**Table 1:** Location and number of samples (n) collected at the various sites.

Code	Site	Country	n	Latitude	Longitude	Collector
DON	Doñana	Spain	36	37.093	–6.292	José L. Arroyo
VAL	Valencia	Spain	23	39.364	–0.346	Juan S. Monrós
SAN	Santo André	Portugal	29	38.099	–8.800	Júlio M. Neto
SAL	Salreu	Portugal	55	40.733	–8.550	Júlio M. Neto
LAN	La Nava	Spain	12	42.066	–4.746	Fernando J. Tazo
FRA	Bretagne	France	35	47.895	–4.352	Bruno Bargain
POL	Biebrza	Poland	2	53.487	17.057	Jacek J. Nowakowski
MUT	Mutěnice	Czech Republic	31	48.897	17.057	Petr Prochazka
NES	Nesyt	Czech Republic	18	48.770	16.700	Petr Procházka, Z. Hubálek
FEN	Fenékpuszta	Hungary	10	46.715	17.246	Norbert Mátrai
IZS	Izsák	Hungary	13	46.783	19.350	Norbert Mátrai
FAR	Farmos	Hungary	11	47.465	19.615	Norbert Mátrai
KAL	Kalimok	Bulgaria	14	44.000	26.260	Pavel Zehtindjiev
SHA	Shabla	Bulgaria	15	43.620	28.560	Mihaela Ilieva
SAR	Saratov	Russia	9	50.825	47.091	Mihaela Ilieva
KRA	Krasnodar	Russia	1	46.130	38.700	[17]
TKH	Lake Tere-Khoi	Russia	2	50.041	95.002	Georgy Semenov
DJO	Djoudj	Senegal	4	16.376	–16.310	Júlio M. Neto
CHA	Lake Chad	Nigeria	10	13.225	13.702	Ulf Ottosson

DNA was extracted from the blood samples using standard phenol-chloroform or salt extraction methods, quantified on a spectrometer and diluted to 5 ng/µl. The complete mitochondrial NADH dehydrogenase subunit 2 gene (ND2; 1041 bp) was amplified and sequenced from both ends using the primers L5215 [16] and H1064 as described by Drovetski et al. [17]. One sequence from Kranosdar, Russia, that was available in GenBank [17] was also used in the haplotype analyses. The samples from Poland, Lake Tere-Khoi, Kranosdar and from the wintering sites were excluded from the population-level analyses.

In addition, eight microsatellite loci were used for population genetic analyses: Ase64 and Ase18 [18], Gf05 [19], Cuμ28 [20], ZL54 [21], Ppi2 [22], Pdoμ5 [23] and Mcyμ4 [24]. These microsatellites were amplified in a standard 10 µl PCR reaction according to the conditions described in Neto et al. [25], mixed and analysed simultaneously on an ABI Prism 3510 Genetic Analyser (Applied Biosystems). Samples that were difficult to score were re-analysed and some new DNA extractions were made in order to minimize errors. Individuals that were genotyped for less than five loci were excluded from subsequent analysis.

### Mitochondrial DNA

The ND2 sequences were aligned and edited in Geneious 5.1 [26] and did not show any evidence for double peaks nor unexpected stop codons suggesting that they were mitochondrial rather than nuclear copies. Molecular diversity indices, Tajima’s ***D*** and Fu’s ***F***s neutrality tests were calculated in Arlequin 3.5.1.2 [27] using a bootstrap procedure with 1000 replicates, whereas the R2 test [28] was calculated in DnaSP software [29]. Mismatch distributions and the time since population expansion (τ = 2 µT) were also calculated in Arlequin and compared with models of sudden population expansion (estimated from 100 bootstrap replicates) using the sum of squared deviations test.

A haplotype network was calculated in TCS [30] using a parsimony algorithm and all the Savi’s Warbler’s sequences. A maximum likelihood tree including all the 57 haplotypes found in Savi’s Warblers and GenBank sequences of its sister species was implemented in PHyML [31] using the best model of molecular evolution as determined by the program jModelTest [32] according to the Akaike information criteria (TrN + I). Maximum parsimony and medium-joining trees calculated in Geneious produced similar results and are not shown.

In addition, we used the coalescent analysis implemented in Beast 1.6.1 [33] to assess whether the ND2 sequences evolved in a clock-like manner, for which the mean and 95% HPD of ucld.stdev was compared to zero in a model where the relaxed uncorrelated lognormal clock was used as prior. Beast was also used to determine the age of the major nodes in two models: (1) a constant population model that includes all Savi’s Warbler haplotypes plus the River Warbler sequences; and (2) a population expansion model, in which all sequences of Savi’s Warblers (including repeated haplotypes) were analysed. For both models, lognormal and strict molecular clocks were implemented with a uniform prior for substitution rates varying from 0.0095 to 0.0115 [34]. A uniform distribution varying from 0.0095 and 0.025 was also used because of the uncertainty associated with the molecular evolution of ND2, which has been reported to be greater than the 2.1% divergence rate of the cytochrome *b* gene [34–37; but see 38]. The models were run multiple times in Beast and evaluated in the program Tracer 1.5, in order to assess the influence of different priors and chain length on the effective sample size and convergence. Final models were run for 20 million generations and sampled every 2000 generations, which resulted in 10000 trees that were summarized and visualized with TreeAnnotator 1.6.1 and FigTree 1.3.1 (discarding the first 10% of trees). Beast was also used to model the demographic changes of the two major clades using the Bayesian skyline analysis [39], for which a chain length of 80 million generations was used for Clade A and 50 million for clades B and B1. Results were analysed and visualized in Tracer.

### Microsatellites

The microsatellites were first evaluated with Micro-checker [40] to determine the presence of null alleles, scoring errors and large allele dropout, and then analysed for Hardy-Weinberg equilibrium (HWE), genetic diversity, population differentiation, AMOVA and isolation-by-distance (Mantel test) in GenA**l**Ex 6.1 [41]. Only 16 out of the 112 tests for HWE were significant (P<0.05). In one population (Valencia), four out of eight loci were not in HWE, which could have been due to the presence of migratory birds in our sample. The fixation index and the estimate of null alleles were low except for Gf05 (Table 2), which we showed earlier to have null alleles [25]. As the analysis excluding this locus and the Valencia population produced similar results, they were used in the final analysis. *D*_est_ [42] was calculated with SMOGD 1.2.5 [43], and all distance matrices were subjected to Principal Component Analyses (PCA) performed in GenA**l**Ex for better visualization of the genetic relationships among populations.

**Table 2:** Number and diversity (±SD) of ND2 sequences used in this study.

Site	n	S	H	Hd	K	π	Fu’s Fs	Tajima’s D	R2	τ
DON	36	26	9	0.818±0.037	8.85±4.17	0.0085±0.0045	4.9^ns^	1.42^ns^	0.17^ns^	19.8
VAL	23	25	8	0.862±0.039	10.67±5.04	0.0103±0.0054	4.7^ ns^	2.16^ ns^	0.21^ns^	20.3
SAN	29	22	7	0.805±0.043	9.38±4.44	0.0090±0.0047	6.7^ns^	2.39^ ns^	0.21^ns^	18.3
SAL	55	24	8	0.750±0.049	9.40±4.38	0.0091±0.0047	9.5^ns^	2.54^ns^	0.20^ns^	18.3
LAN	12	21	5	0.788±0.090	10.55±5.17	0.0101±0.0056	5.0^ns^	2.28^ ns^	0.24^ns^	20.4
FRA	35	7	7	0.797±0.004	1.56±0.95	0.0015±0.0010	–1.0^ns^	–0.23^ns^	0.11^ns^	1.7
MUT	31	18	17	0.897±0.042	1.72±1.03	0.0017±0.0011	–15.3^***^	–2.12^**^	0.04^***^	1.9
NES	18	6	7	0.634±0.127	0.92±0.67	0.0009±0.0007	–4.1^***^	–1.55^*^	0.08^*^	1.0
FEN	10	5	6	0.778±0.137	1.00±0.73	0.0010±0.0008	–3.9^***^	–1.74^*^	0.10^*^	1.3
IZS	13	6	7	0.833±0.086	1.23±0.83	0.0012±0.0009	–4.0^**^	–1.34^ns^	0.09^*^	1.4
FAR	11	8	8	0.891±0.092	1.60±1.03	0.0015±0.0011	–5.4^***^	–1.71^*^	0.08^**^	1.8
KAL	14	4	5	0.802±0.060	1.16±0.80	0.0011±0.0009	–1.2^ns^	–0.24^ns^	0.14^ns^	1.5
SHA	15	11	8	0.791±0.105	1.58±1.00	0.0015±0.0011	–4.0^**^	–2.05^**^	0.09^*^	1.6
SAR	9	5	5	0.833±0.098	2.00±1.24	0.0019±0.0014	–0.9^ns^	0.37^ns^	0.18^ns^	2.7
Clade A	244	49	47	0.807±0.019	1.44±0.882	0.0014±0.00094	–28^***^	–2.37^***^	0.08^***^	1.45
Clade B	90	12	11	0.591±0.0528	1.15±0.753	0.0011±0.00080	–4.5^*^	–1.38^ns^	0.09^ ns^	0.14

Diversity indices were not calculated for populations with very small sample sizes (Biebrza and Krasnodar), nor for wintering sites (Djoud and Lake Chad). ns – non-significant; *** – P<0.001; ** – P<0.01; * – P<0.05.

n – sample size.

S – number of segregating sites.

H – number of haplotypes.

Hd – haplotype diversity.

K – average number of pairwise differences.

**π** – nucleotide diversity.

R2– Ramos-Onsins & Rozas (2002) test.

τ – time of population expansion (τ = 2 µT).

The individual population assignment method implemented in Structure 2.3.3 was also used to detect population structure [44], [45]. This program was run three times for each K = 1 to K = 8 clusters using the admixture model with correlated allele frequencies, sampling location information prior, an initial burn-in of 10^4^ and 10^6^ Markov Chain Monte-Carlo interactions. Multiple runs varying the priors and number of interactions resulted in identical conclusions. The number of clusters was determined using the ad hoc statistics (?K) following Evanno et al. [46] as implemented in the program Structure Harvester 0.3 [47].

### Ethical Treatment of Animals

The collection of all samples was conducted under the licenses required by the corresponding national authorities. Permits were given by the following institutions: Bulgarian Ministry of Environment and Waters (N76/06.07.2006), Conselleria de Medi Ambient, Aigua, Urbanisme i Habitatge, Generalitat Valenciana (440066), Consejería de Medio Ambiente de la Junta de Andalucía (6305), National Environmental, Conservation and Water Inspectorate (14/2436-3/2006 and 14/140/3/2007), Krajsky urad Jihomoravskeho kraje (JMK 31771/2006), Instituto de Conservação da Natureza (73/2005), Centre de Recherches par le Baguage des Populations d’Oiseaux (381805EH44805), State Committee for Hunting and Fishing of Tyva Republic.

## Results

### Mitochondrial DNA

We analysed the complete (1041 bp) ND2 gene of 330 individuals sampled from 17 breeding and two wintering sites, and also included one sequence available in GenBank from Krasnodar, Russia [17]. Overall, there were 68 segregating sites, of which 35 were parsimony informative and 17 caused amino acid changes, resulting in 57 haplotypes (GenBank accession numbers: JQ996503 to JQ996558, and A382380 [17]). The overall haplotype diversity (Hd) was 0.868±0.011, the nucleotide diversity (π) was 0.00784±0.00037, and the average number of nucleotide differences (k) was 8.158±3.794. Population-specific diversity indices are shown in Table 2.

**Figure 1:**
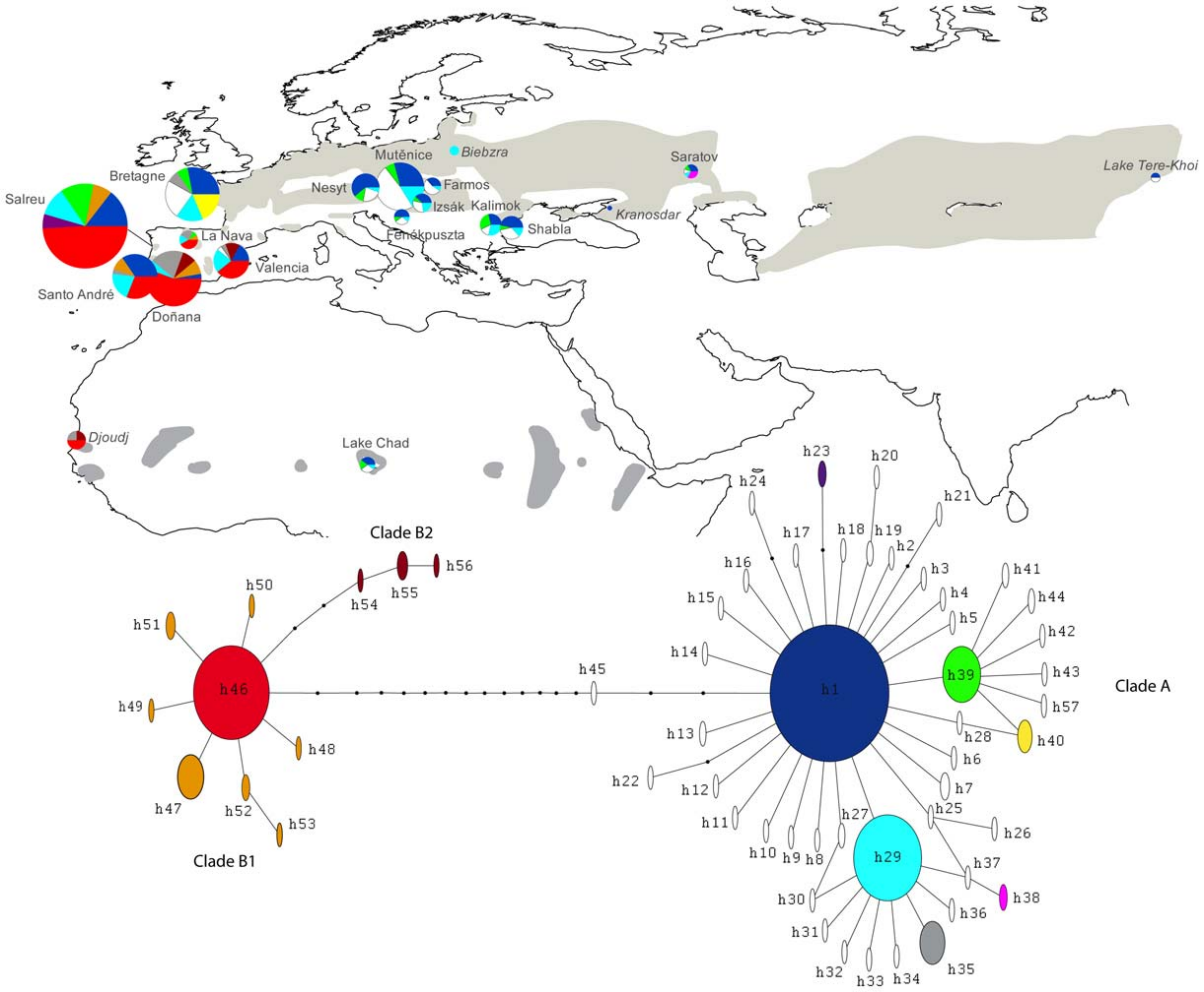
Location of the sampling sites, minimum-spanning network and frequency of all 57 ND2 haplotypes found in Savi’s Warblers. Circles are proportional to the sample size except for the locations in italic, in which the size was trebled. The grey shade represents the approximate breeding and wintering (in Africa) distributions of this species.

**Figure 2:**
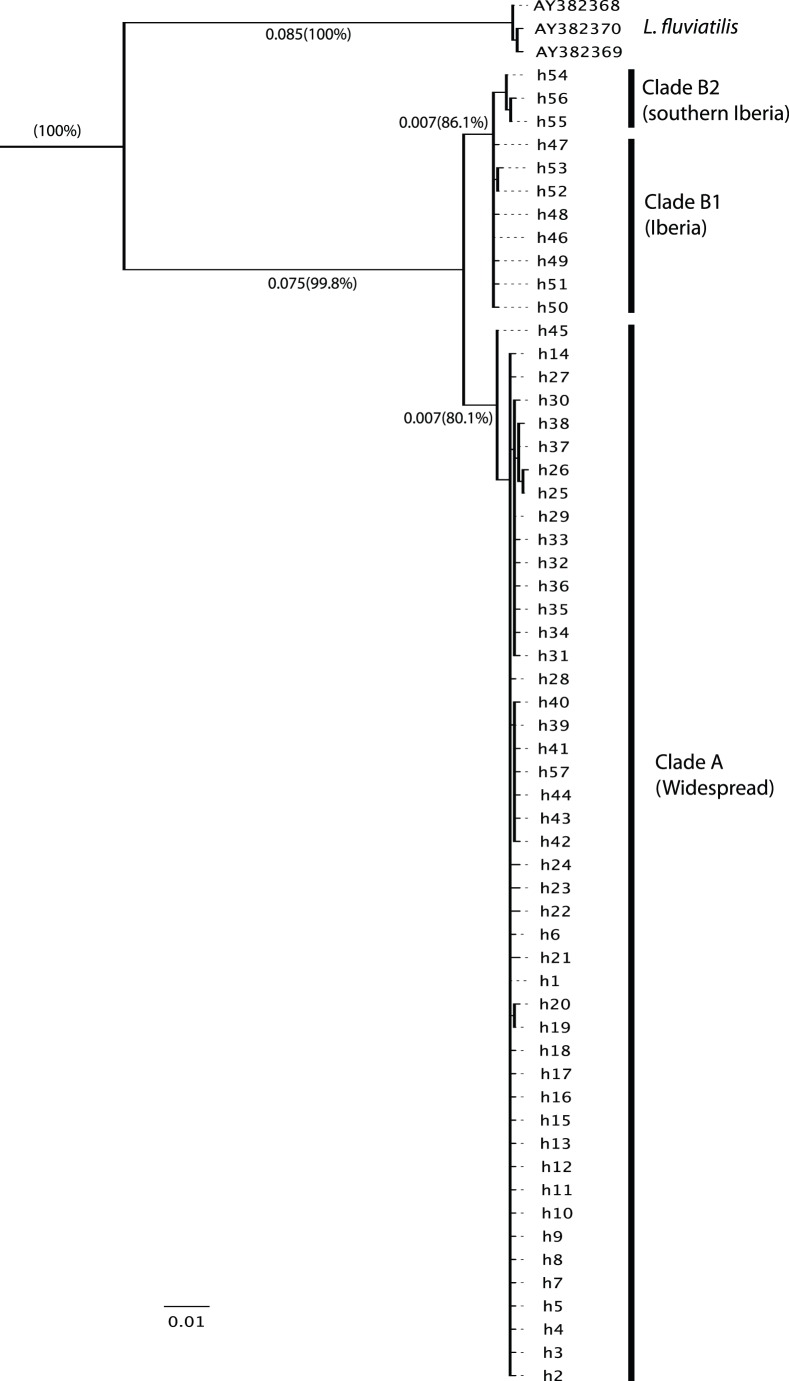
Maximum likelihood tree. of all the 57 haplotypes found in Savi’s Warbler plus its sister species *Locustella fluviatilis,* based on the model of molecular evolution TrN+I. Numbers indicate the branch lengths in substitutions per site and, between parenthesis, the bootstrap support (1000 replicates).

The haplotype network (Figure 1) and the maximum likelihood tree (Figure 2) show that Savi’s Warbler’s haplotypes are divided into two distinctive clades, which differ by 14–25 nucleotide substitutions (i.e. 1.3–2.4% of uncorrected divergence). The maximum–likelihood tree (Figure 2) indicates that the root was located between the two Savi’s warbler clades, and that Savi’s Warbler differ from its sister species, the River Warbler, by 9.2% of uncorrected divergence (see also [17], [48]). This strongly suggests that two populations of Savi’s Warblers evolved independently for a considerable period of time.

**Figure 3:**
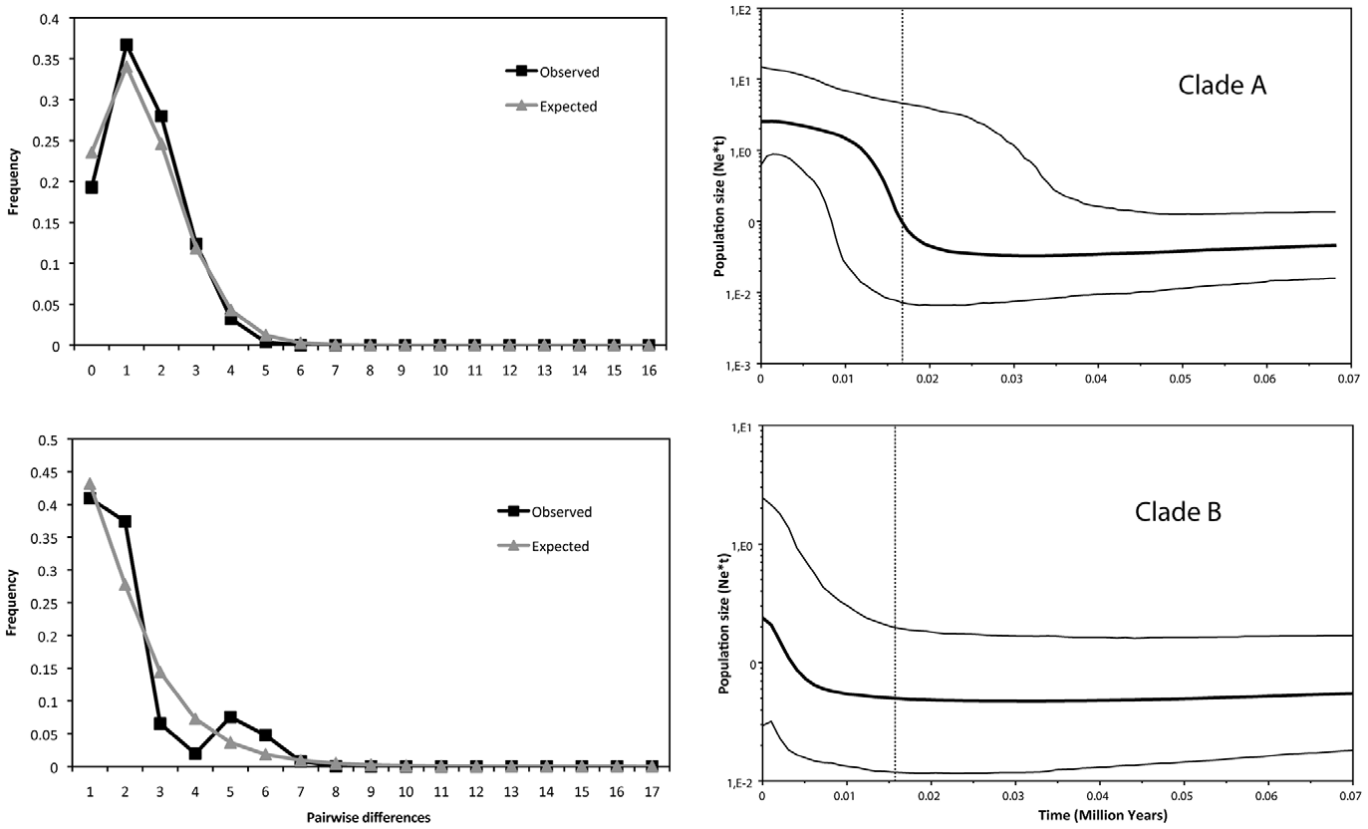
Mismatch distribution and Bayesian skyline analyses. Observed mismatch distributions (left) for clade A (above) and clade B (below) and Bayesian skylines showing the median population size through time for each clade as well as the upper and lower 95% HPD. The comparison with the expected distributions for sudden population growth (grey lines) is not significantly different for clade A (Sum of Squared Deviations = 0.0031, P = 0.08; τ = 1.45), but is significantly different for clade B (SSD = 0.0151, P = 0.01; τ = 0.14).

The two Savi’s Warbler clades have different structures and diversity, as well as geographical distribution (Figure 1, Table S1). The most diverse clade (A) occurs throughout the entire sampling area and has a star shape with three very common, widespread, haplotypes (h1, h29, h39) in the centre and many rare haplotypes differing by only 1–3 mutations from the most frequent haplotype (h1). Some geographical structure is evident (Figure 1, Table S1): the closest haplotype to the root (h45) was found in Shabla, Bulgaria, whereas the tips of the clade include haplotypes that were found only in Russia (h36, h38), France and Iberia (h35), France (h15, h26, h40) and Iberian Peninsula (h23) (Figure 1, Table S1). Neutral tests (Table 2) and mismatch distribution (Figure 3) indicate that clade A was subject to a population bottleneck followed by rapid expansion or to a selective sweep. The skyline Bayesian analysis also indicates a population expansion of this clade, the timing of which corresponds approximately with the peak of the last glaciation (Figure 3).

Clade B, which occurred exclusively in the Iberian Peninsula, has a lower diversity and is more structured than clade A. Indeed, three of the haplotypes (h54, h55, h56– hereafter clade B2) that differ from the most frequent one (h46) by 3–5 substitutions are clustered together in the minimum spanning network as well as in the ML tree (Figures 1 and 2). These three haplotypes occurred only at two sites in the southern part of the Iberian Peninsula (Doñana and Valencia), despite the relatively large sample size in other parts of Iberia (e.g. Salreu), suggesting that there is geographical structure within this region. The statistical tests of neutrality (Table 2), mismatch distribution and skyline Bayesian analysis (Figure 3) do not indicate population growth of clade B, except perhaps a very recent and limited growth. When clade B2 is excluded because of the confounding effect of geographic structure, the neutrality tests applied to clade B1 remains non-significant (τ = 0.612, R2 = 0.105, P = 0.1; Fu’s ***F***s =  –0.009, P = 0.5; Tajima’s ***D*** = 0.036, P = 0.5), and similar results are obtained for the mistmatch and Bayesian skyline analyses.

**Figure 4:**
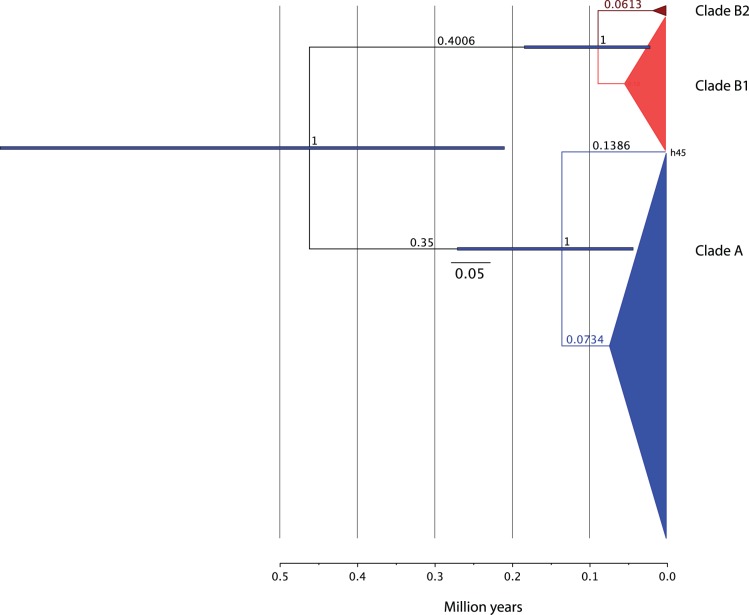
Maximum clade credibility tree. Results from a Beast run using all Savi’s Warblers sequences, a population expansion model and strict clock (uniform distribution 0.0095–0.025 substitutions per million years). Horizontal blue bars represent the 95% HDP of the age of major nodes. Numbers indicate node posterior probability and branch lengths.

The coalescent analysis implemented in Beast produced a maximum clade credibility tree similar to the ML tree, and full posterior support for the nodes splitting the clades A-B, and B1–B2 (Figure 4 and Figure S1). As the standard deviation of substitution rates (ucld.stdev) of the models using relaxed uncorrelated lognormal clock was nearly zero, the strict molecular clock could not be rejected for our data. When all the sequences were used in an expansion growth model and a strict clock (varying uniformly from 0.0095 to 0.025), clades A and B diverged in the early to mid-Pleistocene (0.49 MYBP; 95% HPD: 0.22–0.87), whereas clades B1 and B2 diverged c. 0.082 MYBP (95% HPD: 0.01–0.18), which corresponds approximately to the beginning of the last glacial maxima (Figure 4). Beast runs including three River Warbler sequences and all 57 Savi’s Warbler’s haplotypes using strict clock and constant populations indicate that the divergence of Savi’s Warbler from its sister species occurred in the Pliocene (5.5 MYBP; 95% HPD: 2.55–9.31), and the split between clades A and B (0.69 MYBP; 95% HPD: 0.31–1.19), and between clades B1 and B2 (0.147 MYBP; 95% HPD: 0.03–0.31) were slightly older than in the population growth model (Figure S1).

If a molecular rate of 2.1% is used, as in other studies (Weir & Schluter, 2008; McCormack et al., 2011), the divergence between Savi’s and River Warblers occurred 8.34 MYBP, whereas between clades A and B and B1/B2 was 1.07 and 0.23 MYBP, respectively. The expansion models with this molecular rate yielded divergence dates for clades A/B and B1/B2 of 0.79 and 0.13 MYBP, respectively.

Neutral tests and mismatch analysis of the sampled populations show strong evidence for population expansion in the Czech Republic, Hungary and Shabla, Bulgaria, but not for the populations in the Iberian Peninsula, France or Russia (Table 2).

**Figure 5:**
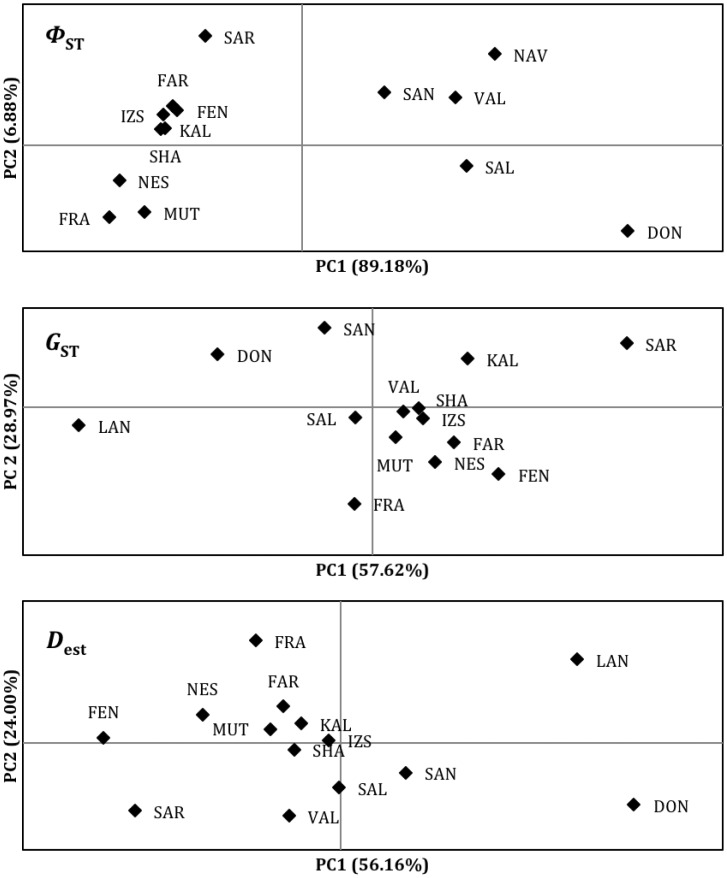
Principal component analysis plots based on the genetic distance matrices (*Φ*_ST_, *G*_ST_ and Jost’s *D*_est_) presented in **Table 2**. Axis titles show the percentage of explained variation.

**Figure 6:**
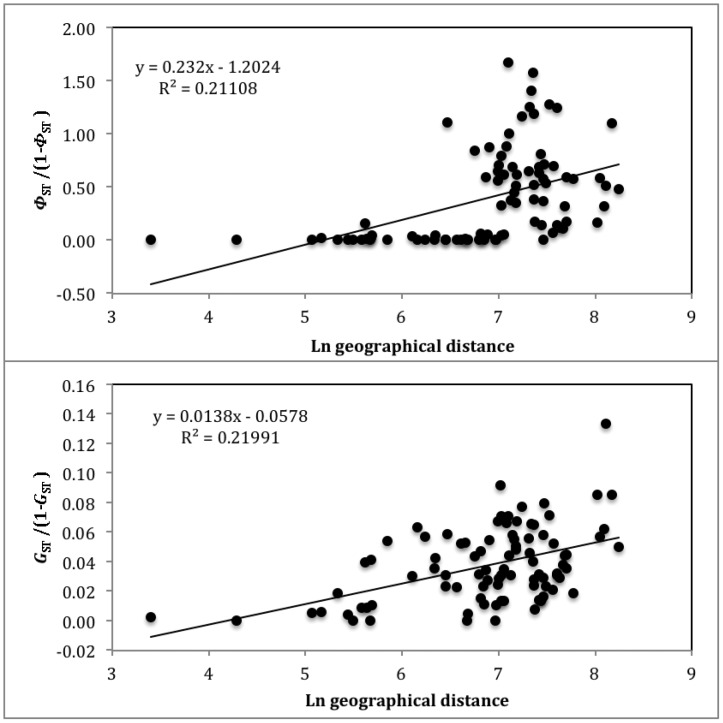
Isolation-by-distance. Analyses including all sampling sites are shown. Mantel tests were significant for both *Φ*_ST_ (P = 0.001) and *G*_ST_ (P = 0.001).

The overall *Φ*_ST_ was highly significant (0.35, P<0.001) and varied between zero and 0.63 (Doñana vs. France). It was particularly high between the Iberian sites and the remaining populations, and Russia also stands out being significantly different from the remaining populations (Table 3, Figure 5). The AMOVA shows that the among-group variance component is highly significant (Table 4). When the populations are divided into groups, Iberian versus the remaining populations explain a greater proportion of the among-group variance component than any other group combination (Table 4), in agreement with the distribution of the different clades. Despite this obvious genetic break, IBD was observed in mitochondrial DNA when all populations were considered (Figure 6). Within Iberia, IBD was not significant (Mantel test: Ln geographical distance = –0.08*Φ*_ST_/(1-*Φ*_ST_)+0.519; R^2^ = 0.325; P = 0.06), but the remaining sites still showed a very significant pattern of IBD (Mantel test: Ln geographical distance = 0.04*Φ*_ST_/(1-*Φ*_ST_)+0.216; R^2^ = 0.345; P<0.008).

**Table 3:** Genetic distances between populations: *Φ*_ST_ and *G*_ST_ and Jost’s *D*_est_.

*Φ*_ST_	DON	VAL	SAN	SAL	LAN	FRA	MUT	NES	FEN	IZS	FAR	KAL	SHA
VAL	0.042												
SAN	**0.134**	0.000											
SAL	0.037	0.000	0.020										
NAV	0.000	0.000	0.000	0.000									
FRA	**0.625**	**0.465**	**0.359**	**0.456**	**0.526**								
MUT	**0.612**	**0.446**	**0.337**	**0.441**	**0.501**	0.030							
NES	**0.584**	**0.408**	**0.307**	**0.412**	**0.469**	0.056	0.006						
FEN	**0.538**	**0.341**	**0.247**	**0.371**	**0.377**	0.049	0.000	0.000					
IZS	**0.556**	**0.366**	**0.271**	**0.392**	**0.407**	0.042	0.000	0.039	0.000				
FAR	**0.543**	**0.349**	**0.257**	**0.381**	**0.382**	0.048	0.000	0.008	0.000	0.000			
KAL	**0.560**	**0.371**	**0.278**	**0.393**	**0.416**	0.000	0.000	0.014	0.014	0.000	0.000		
SHA	**0.555**	**0.366**	**0.267**	**0.386**	**0.409**	**0.067**	0.000	0.007	0.000	0.000	0.000	0.019	
SAR	**0.523**	**0.322**	**0.239**	**0.369**	**0.339**	**0.144**	**0.098**	**0.242**	**0.146**	**0.103**	**0.121**	**0.126**	**0.148**

*G*_ST_	DON	VAL	SAN	SAL	LAN	FRA	MUT	NES	FEN	IZS	FAR	KAL	SHA
VAL	**0.041**												
SAN	**0.038**	**0.050**											
SAL	**0.029**	**0.022**	**0.039**										
LAN	**0.034**	**0.059**	**0.054**	**0.051**									
FRA	**0.066**	**0.051**	**0.063**	**0.042**	**0.055**								
MUT	**0.039**	**0.013**	**0.046**	**0.013**	**0.042**	**0.023**							
NES	**0.061**	**0.030**	**0.052**	**0.027**	**0.062**	**0.015**	0.002						
FEN	**0.072**	**0.023**	**0.066**	**0.033**	**0.084**	**0.027**	0.000	0.004					
IZS	**0.044**	**0.016**	**0.030**	**0.024**	**0.055**	**0.030**	0.000	0.010	0.005				
FAR	**0.061**	**0.023**	**0.048**	**0.034**	**0.063**	0.013	0.008	0.009	0.018	0.000			
KAL	**0.067**	**0.034**	**0.027**	**0.053**	**0.074**	**0.055**	**0.031**	**0.045**	**0.050**	**0.030**	**0.022**		
SHA	**0.030**	**0.018**	**0.028**	**0.014**	**0.050**	**0.021**	0.000	0.010	0.011	0.005	0.000	0.006	
SAR	**0.078**	**0.048**	**0.058**	**0.054**	**0.117**	**0.079**	**0.037**	**0.042**	**0.043**	**0.028**	**0.031**	0.014	0.008

*D*_est_	DON	VAL	SAN	SAL	LAN	FRA	MUT	NES	FEN	IZS	FAR	KAL	SHA
VAL	0.069												
SAN	0.042	0.078											
SAL	0.047	0.034	0.029										
LAN	0.049	0.100	0.070	0.075									
FRA	0.127	0.092	0.088	0.077	0.087								
MUT	0.093	0.039	0.069	0.020	0.070	0.042							
NES	0.122	0.051	0.071	0.050	0.095	0.023	0.001						
FEN	0.153	0.054	0.090	0.033	0.138	0.045	0.002	0.005					
IZS	0.049	0.017	0.014	0.012	0.021	0.027	0.000	0.001	0.017				
FAR	0.102	0.035	0.049	0.059	0.024	0.019	0.000	0.003	0.039	0.000			
KAL	0.106	0.052	0.027	0.033	0.032	0.024	0.010	0.016	0.031	0.000	0.000		
SHA	0.066	0.034	0.050	0.015	0.076	0.030	0.000	0.011	0.008	0.000	0.000	0.000	
RUS	0.142	0.045	0.076	0.065	0.151	0.101	0.038	0.024	0.036	0.000	0.000	0.003	0.000

Significant genetic distances (P<0.05; 1000 bootstrap permutations) are indicated in bold except for *D*_est_, for which no statistical test is available.

**Table 4:** Analysis of molecular variance (AMOVA) based on pairwise differences between ND2 sequences and microsatellites.

ND2 pairwise differences							
Source of variation	d.f.	SS	Variance	% var.	Φ-stat.	P	
Among Populations	13	455.477	1.49127	35.0	0.35	<0.001	
Within Populations	297	822.073	2.76792	65.0			
Total	310	1277.550	4.25920				

ND2 pairwise differences; Iberia vs. other populations							
Source of variation	d.f.	SS	Variance	% var.	Φ-stat.	P	
Among Regions	1	412.188	2.62529	48.3	0.483	<0.001	
Among Populations	12	43.289	0.04005	0.7	0.014	0.077	
Within Populations	297	822.073	2.76792	50.9	0.491	<0.001	
Total	310	1277.550	5.43320				

Microsatellites							
Source of variation	d.f.	SS	MS	Variance	% var.	G-stat.	P
Among Populations	13	86.348	6.642	0.088	3.6	0.036	0.001
Among Individuals	316	830.201	2.627	0.267	10.9	0.113	0.001
Within Individuals	330	691.000	2.094	2.094	85.5	0.145	0.001
Total	659	1607.548		2.448			

Microsatellites; Iberia vs central Europe vs Russia							
Source of variation	d.f.	SS	MS	Est. Var.	%	G-stat	P
Among Regions	2	29.958	14.979	0.045	1.7	0.026	0.001
Among Populations	11	66.947	6.086	0.069	2.5	0.042	0.001
Among Individuals	316	928.652	2.939	0.306	11.1	0.116	0.001
Within Individuals	330	768.500	2.327	2.327	83.9	0.153	0.001
Total	659	1793.558		2.748			

### Microsatellites

Only 14 out of the 112 tests for HWE were significant. In one population (Valencia) three out of eight loci were not in HWE, and one locus (Pdoμ5) was not in HWE in six out of 14 populations. However, the fixation index was very low for most loci except Gf05 (Table S2), probably due to the presence of null alleles [25]. As the analyses excluding Gf05 resulted in similar conclusions, we present the results obtained from the complete dataset.

The mean unbiased expected heterozygosity varied between 0.53 at La Nava to 0.68 at Mutěnice (Table S2), but was generally similar among populations. Population comparisons resulted in a highly significant but moderate *G*_ST_ (0.036; P<0.001), varying between zero and 0.117 (La Nava vs Saratov; Table 3). *G*_ST_ was particularly high among the Iberian populations, but France, Saratov and Kalimok were also significantly different from most other populations (Table 3, Figure 5). In contrast to the mtDNA, Valencia (and to some extent Salreu) lies very close to the central European populations in the PCA based on the *G*_ST_ matrix (Figure 5), suggesting that a genetic break in the Pyrenees region does not occur in nuclear DNA. However, three groups (Iberia, Central Europe, Saratov) produced the greatest genetic variance in AMOVA (Table 4). Jost’s *D*_est_ genetic distances produced results broadly similar to *G*_ST_, although Fenékpuszta, rather than Kalimok, seemed to be comparatively distant from the central European populations, and Salreu and Valencia were relatively more distinct (from central European sites) than in the *G*_ST_ analysis (Table 3, Figure 5).

### Microsatellites

Only 14 out of the 112 tests for HWE were significant. In one population (Valencia) three out of eight loci were not in HWE, and one locus (Pdoμ5) was not in HWE in six out of 14 populations. However, the fixation index was very low for most loci except Gf05 (Table S2), probably due to the presence of null alleles [25]. As the analyses excluding Gf05 resulted in similar conclusions, we present the results obtained from the complete dataset.

The mean unbiased expected heterozygosity varied between 0.53 at La Nava to 0.68 at Mutěnice (Table S2), but was generally similar among populations. Population comparisons resulted in a highly significant but moderate *G*_ST_ (0.036; P<0.001), varying between zero and 0.117 (La Nava vs Saratov; Table 3). *G*_ST_ was particularly high among the Iberian populations, but France, Saratov and Kalimok were also significantly different from most other populations (Table 3, Figure 5). In contrast to the mtDNA, Valencia (and to some extent Salreu) lies very close to the central European populations in the PCA based on the *G*_ST_ matrix (Figure 5), suggesting that a genetic break in the Pyrenees region does not occur in nuclear DNA. However, three groups (Iberia, Central Europe, Saratov) produced the greatest genetic variance in AMOVA (Table 4). Jost’s *D*_est_ genetic distances produced results broadly similar to *G*_ST_, although Fenékpuszta, rather than Kalimok, seemed to be comparatively distant from the central European populations, and Salreu and Valencia were relatively more distinct (from central European sites) than in the *G*_ST_ analysis (Table 3, Figure 5).

We also carried out an AMOVA comparing microsatellite diversity between individuals from the Iberian Peninsula that carried mtDNA haplotypes of clade A versus those with clade B. The percentage of variation between these two “populations” was very low (1%), but significant (*G*_ST_ = 0.009; P = 0.002).

**Figure 7:**
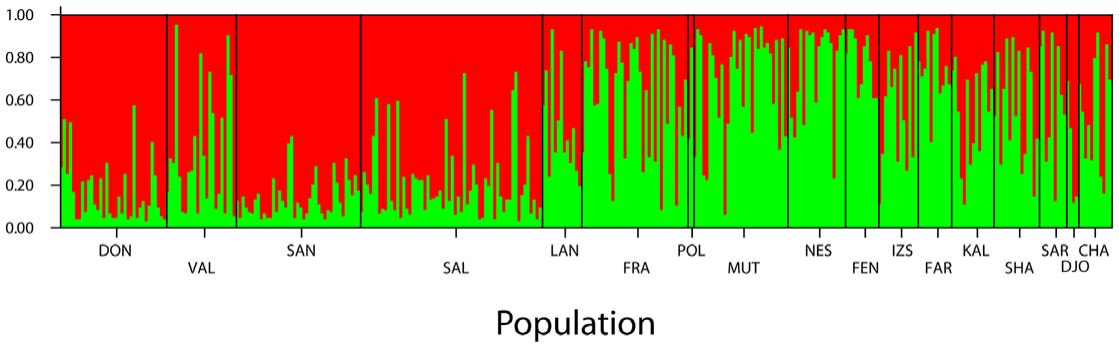
Results from STRUCTURE analysis. Vertical bars represent the probability of belonging to each cluster (K = 2) for each individual tested.

The individual-based analysis implemented in Structure resulted in two groups, which had greater ΔK and L(K) [46]. These groups separated most Iberian birds from those sampled in central and eastern European sites (Figure 7), and agrees well with the mitochondrial DNA, despite La Nava and Valencia including a considerable number of individuals more similar to those from central Europe. IBD was apparent from both measurements of genetic distance between populations (Figure 6), as was found for mtDNA. However, within Iberia, IBD was not significant (Mantel test: Ln geographical distance = –0.004*Φ*_ST_/(1-*Φ*_ST_)+0.066; R^2^ = 0.009; P = 0.407) but, as with mtDNA, the remaining sites showed a highly significant pattern of IBD (Mantel test: Ln geographical distance = 0.011*Φ*_ST_/(1-*Φ*_ST_)+0.052; R^2^ = 0.366; P<0.001).

The two wintering populations show evidence for little mixing: the birds from Senegal belong to the Iberian populations, both according to the mtDNA (Figure 1, Table S1) and the microsatellites (Figure 7), whereas those from Lake Chad seem to belong to central Europe.

## Discussion

Phylogeographic analysis has revealed common patterns in the genetic structure of numerous species, suggesting that at least some historical events were extremely influential at the community level [1], [2], [7]. But there are also species-specific idiosyncrasies that are difficult to explain. These particularities may depend on the interaction of historical events with the species behaviour and ecology, for instance with the dispersal capability, philopatry, habitat, foraging niche etc. In order to understand the influence of these factors on the genetic structure, biogeography and speciation, multiple species need to be studied in detail and compared [4].

Our study on a migratory, habitat-specialist bird revealed considerable genetic structure, especially within the populations belonging to the nominate subspecies. It was shown that Savi’s Warblers have two major mitochondrial lineages that originated in the early or middle Pleistocene and evolved separately for a period of time spanning several glacial and interglacial periods. The distribution of these lineages and their genealogy is consistent with the existence of two refugia: one in Iberia and the other in the Balkan region; and the latter was the source of individuals that colonized most of the Palaearctic. Our results indicate that the two clades met only very recently, rather than during previous interglacial periods, as mismatch and Bayesian skyline analyses are consistent with population expansion only after the last glacial maxima. Our results also support the observation of eastern populations expanding earlier and more extensively than western populations, either because there are fewer barriers to dispersal, larger populations, larger patches of suitable habitat and/or the climate ameliorated comparatively earlier in the east than in western Mediterranean [2], [6].

In Savi’s Warblers, the post-glacial colonization proceeded towards Europe and Asia from the Balkans and resulted in an IBD pattern [49]. The genetic structure and IBD observed in mtDNA seem to be supported by the nuclear markers, even though microsatellites are not ideal for phylogeographic inference due to lack of genealogical information and homoplasy [50]. This IBD pattern is absent from the Iberian Peninsula and stronger at the remaining sites, and seems to have occurred as a direct consequence of the range expansion, which was slow enough for new mutations to appear and generate allele frequency gradients [49]. Allele surfing also seems to have occurred [49]; for instance, haplotype 35, which belongs to the eastern clade (represented in grey colour in Figure 1) may have appeared somewhere in France, but reached greater frequencies in eastern Iberian Peninsula.

The divergence among populations is much smaller in microsatellite than in mtDNA, but this is expected given the smaller effective population size of mtDNA and the large variation of microsatellites [42], [51]. In addition, the measurements of genetic distance among populations are not comparable, as *Φ*_ST_ takes into account the distance between haplotypes, whereas *G*_ST_ only considers differences in allele frequencies among populations. These differences in the measurement of genetic distance (as well as homoplasy and greater effective population size of microsatellites) might be the reason for some Iberian sites appearing much closer to central European sites in nuclear than in mitochondrial DNA (Figure 5). Nevertheless, as with mtDNA, AMOVA and STRUCTURE analyses of microsatellites give some support to the existence of a genetic break in the Pyrenees region, despite the extensive introgression of mitochondrial and nuclear genes from Central Europe to Iberia. In addition, the French sample is not close to the Iberian sites in any analyses (Figure 5). However, nuclear sequencing and a more extensive sampling around the Pyrenees should be undertaken in order to assess the differences in the relative introgression of nuclear and mitochondrial DNA and the exact location of the genetic breaks.

Although the sample size of *L. l. sarmatica* and *L. l. fusca* is small, it seems that these subspecies evolved recently and do not represent deep evolutionary units, which is a pattern found in most Palaearctic bird species [51]. Pale plumage in eastern birds is a recurrent feature in many birds (e.g. *Acrocephalus scirpaceus fuscus*, *A. arundinaceus zarudnyi*; [11], [52]), strongly suggesting that natural selection is involved in the evolution of this trait. Our results support the role of post-glacial expansion into new environments, in opposition to evolution at glacial refugia, as a promoter of intraspecific morphological diversification and, eventually, speciation [49], [52], [53].

Overall, our results agree well with other phylogeography studies from Europe [1], [2]. However, there are three noteworthy observations. First, given that Savi’s Warblers have a high dispersal capability, it is surprising that birds from the Iberian Peninsula did not disperse much north of the Pyrenees region. It is common for the Alps to be a barrier for dispersal of birds that lived in the Italian Peninsula during the last glaciations, but the Pyrenees usually only constitutes a barrier for amphibians, reptiles and some mammals, which have much lower dispersal capabilities [2], [8]. To our knowledge, only three species of birds showed evidence for intraspecific phylogeographic breaks in the Pyrenees region: the Tawny Owl (*Strix aluco*), the Green Woodpecker (*Picus viridis*) and the Red-legged Partridge (*Alectoris rufa*), all of which are sedentary species [54]–[56]. The migratory Iberian Chiffchaffs (*Phylloscopus ibericus*) also meet with European Chiffchaffs (*P. collybita*) in the Pyrenees, but their divergence is much older than the species considered earlier, as the mtDNA divergence between these species is 4.6% [57]. Evidently, the Pyrenees did not constitute a physical barrier for Savi’s Warblers, as the eastern birds crossed the Pyrenees and expanded into Iberia. The genetic break found in the Pyrenees region most probably results from the striking demographic differences between the two mitochondrial clades. It is possible that the population size was very small in the Iberian Peninsula because there is comparatively little habitat available for wetland species (there are currently very few lakes and during the glacial periods the situation was probably worse as the climate was much dryer). In fact, as most reed beds are located in estuaries or coastal lagoons in the Iberian Peninsula, the rapid rise in sea level after the last glaciation could have caused temporary habitat loss and population decline in this habitat specialist, which then failed to colonize Europe. If this is the case, other reed bed specialists are expected to show similar phylogeographic patterns to the Savi’s Warbler. However, very few reed bed species have been analysed so far. In the Great Reed Warbler (*Acrocephalus arundinaceus*), Iberian birds did colonize Europe after the last glaciation (Hansson et al., 2008). But the pattern is still not clear for other species like the Reed Bunting (*Emberiza schoeniclus*), for which there are two near-endemic Iberian subspecies [58], and the Reed Warbler (*Acrocephalus scirpaceus*), in which Iberian and African birds seem to be genetically distinct from the rest of the Palaearctic (Hamid Rguibi-Idrissi, Urban Olsson, Frédéric Jiguet et al., in prep) [59]. Genetic studies focusing on reedbed fauna and flora are needed for comparative phylogeography and the conservation of this threatened habitat and its specialized species.

Second, despite the long period of isolation between the two refugial populations, post-glacial expansion resulted in complete introgression in the Iberian Peninsula, rather than in a suture or hybrid zone. Hybrid zones can occur among populations that are much more closely related than Savi’s Warbler’s populations, as in the case of Willow Warblers (*Phylloscopus trochilus*), whose expansion around the Baltic led to the evolution of distinct migratory directions and a hybrid zone in mid-Scandinavia [60]. However, suture or hybrid zones may only occur between populations that were subject to adaptive evolution and either met at a transition zone or developed some level of reproductive isolation. If the environment at the two refugia was similar, or if the population size was so small that drift was more important than selection, birds from both populations would be able to interbreed and introgress. The role of demographics could also have been important in causing the introgression, as the demographic pressure of the expanding population could perhaps overtake ecological disadvantages, although this has not been studied adequately. It is not possible to determine whether Iberian Savi’s Warblers were morphologically or ecologically different from those of the Balkans, as nuclear introgression is currently very extensive in Iberia. We did find a significant *G*_ST_ between birds with mtDNA belonging to the two clades within Iberia, which could perhaps reflect the asymmetrical introgression from the local birds to the expanding population expected from theory [49], but the difference was very small.

Third, genetic structure within the Iberian clade is consistent with the existence of two refugia within the Iberian Peninsula during the last glaciation. Refugia within refugia have been found in amphibians, reptiles and small mammals in Iberia, in which mountain ranges and, sometimes, major rivers seemed to constitute insurmountable barriers for dispersal [8]. But this has only recently been found in birds, in the sedentary Red-legged Partridge [55]. It is possible that clade B2 originated in a refuge located in North Africa and then expanded to southern Iberia, which could perhaps still be considered refugia within refugia due to the geographic proximity, but we were unable to test this as we lack of samples from Africa. Green Woodpeckers from North Africa (Levaillant’s Woodpecker *Picus vaillantii*) are distinct from Iberian birds [56], and the same happens with the Atlas Flycatcher (*Ficedula speculigera*) [61], but these species differentiated much earlier in association with vicariance events unrelated to the glacial cycles. To our knowledge, the only bird species showing recent divergence between Iberia and North Africa is the sedentary Dupont’s Lark (*Chersophilus duponti*), which seems to have had two isolated refugia in these regions during the last glaciation [62]. Additional sampling would be very useful to determine the exact distribution of B2 haplotypes. Nevertheless, this is the first migratory bird species for which there is evidence of refugia within refugia in the Iberian Peninsula or Iberia/North Africa. Given Savi’s Warbler’s naturally fragmented distribution, the cost of dispersal might be high in this reed bed specialist, and the pattern found could have resulted from strong philopatry.

This study shows that Savi’s Warbler’s populations of the Iberian Peninsula deserve a special conservation effort, as they hold a high genetic variation that includes all the western haplotypes, and should be considered a distinct management unit [63]. This is especially so given their current vulnerability, which results from small population size and restricted distribution [13], [14].

Finally, genetic variation within the Savi’s Warbler seems to be large enough to determine broad patterns of migratory connectivity [64]. Despite the small sample size from wintering areas, birds wintering in Senegal clearly originated from the Iberian Peninsula, whereas those of Lake Chad probably migrated from central/eastern Europe or Asia. Genetic variation among populations within this species might be very useful to determine the origin of wintering birds, especially given that the probability of recapture of marked birds between Europe and Africa is very low, and this could have important conservation implications [64].

## Supporting Information

Table S1 **Haplotype frequencies per population.** Colours indicate the haplotypes belonging to clades A (blue), B1 (red) and B2 (brown). Population names are as described in Table 1. GenBank accession numbers are AY382380 for h1 [17], and JQ996503 to JQ996558 for the remaining haplotypes (this study).
(EPS)

Table S2 **Microsatellite statistics.** Mean and standard error (SE) of microsatellite statistics across populations (above) and across loci (below): number of individuals (n), number of alleles (Na), effective number of alleles (Ne) observed heterozygosity (Ho), expected heterozygosity (He), unbiased He (UHe) and fixation index (F). Population names are as described in Table 1.
(EPS)

Figure S1 **Maximum clade credibility tree.** Result of a coalescent analysis implemented in BEAST with strict clock (uniform distribution 0.0095–0.025 substitutions per million years) and constant population size as priors. Horizontal blue bars represent the 95% HDP of the age of major nodes; branch length and posterior probability of the nodes are also indicated.
(EPS)
